# Fiscal and Policy Implications of Selling Pipe Tobacco for Roll-Your-Own Cigarettes in the United States

**DOI:** 10.1371/journal.pone.0036487

**Published:** 2012-05-02

**Authors:** Daniel S. Morris, Michael A. Tynan

**Affiliations:** 1 Tobacco Prevention & Education Program, Health Promotion & Chronic Disease Prevention, Oregon Health Authority, Portland, Oregon, United States of America; 2 Office on Smoking and Health, National Center for Chronic Disease Prevention and Health Promotion, Centers for Disease Control and Prevention, Atlanta, Georgia, United States of America; The University of Hong Kong, Hong Kong

## Abstract

**Background:**

The Federal excise tax was increased for tobacco products on April 1, 2009. While excise tax rates prior to the increase were the same for roll-your-own (RYO) and pipe tobacco, the tax on pipe tobacco was $21.95 per pound less than the tax on RYO tobacco after the increase. Subsequently, tobacco manufacturers began labeling loose tobacco as pipe tobacco and marketing these products to RYO consumers at a lower price. Retailers refer to these products as “dual purpose" or “dual use" pipe tobacco.

**Methods:**

Data on tobacco tax collections comes from the Alcohol and Tobacco Tax and Trade Bureau. Joinpoint software was used to identify changes in sales trends. Estimates were generated for the amount of pipe tobacco sold for RYO use and for Federal and state tax revenue lost through August 2011.

**Results:**

Approximately 45 million pounds of pipe tobacco has been sold for RYO use from April 2009 to August 2011, lowering state and Federal revenue by over $1.3 billion.

**Conclusions:**

Marketing pipe tobacco as “dual purpose" and selling it for RYO use provides an opportunity to avoid paying higher cigarette prices. This blunts the public health impact excise tax increases would otherwise have on reducing tobacco use through higher prices. Selling pipe tobacco for RYO use decreases state and Federal revenue and also avoids regulations on flavored tobacco, banned descriptors, prohibitions on shipping, and reporting requirements.

## Introduction

Increasing the price of tobacco products is an evidence-based intervention that prevents initiation of tobacco use among adolescents and young adults, reduces consumption of tobacco, and increases quit attempts [1]–[4]. Excise taxes are the most direct way for governments to increase the price of tobacco products [2], [4]. However, tobacco users may seek sources of lower priced tobacco products in response to a price increase instead of quitting tobacco use or reducing consumption, undermining the public health impact of the tax increase [5]. Strategies employed to avoid paying higher prices include, but are not limited to, crossing state borders to purchase products in states with a lower excise tax; purchasing no-to-low taxed products over the internet or at Native American reservations; purchasing no-to-low taxed products on the black market; switching to discount brands; or making roll-your-own (RYO) cigarettes [5]–[9]. Tobacco manufacturers have also reformulated or re-labeled products to capitalize on disparities between tax rates on different types of tobacco products and minimize the impact taxes have on product prices [10].

The Federal excise tax for tobacco products was increased on April 1, 2009 (Table 1) [11]. While the tax on cigarettes, snuff and pipe tobacco was increased by 158%, the tax on small cigars and RYO tobacco increased by a greater amount to make those rates equivalent to the tax levied on cigarettes [11]. Previously, the excise tax rates for RYO and pipe tobacco were the same, but after the increase, the tax on pipe tobacco was $21.95 per pound less than the tax on RYO tobacco [11].

**Table 1:** Change in federal excise tax for all tobacco products, April 1, 2009.

Product	Tax Prior to April 1, 2009	Tax as of April 1, 2009
Cigarettes	$19.50 per 1,000	$50.33 per 1,000
Small Cigars	$1.83 per 1,000	$50.33 per 1,000
Large Cigars	20.72% of sales price, $0.05 maximum per cigar	52.75% of sales price, $0.4026 maximum per cigar
Snuff	$0.59 per pound	$1.51 per pound
Pipe Tobacco	$1.01 per pound	$2.83 per pound
Roll Your Own	$1.01 per pound	$24.78 per pound

After this tax disparity developed, RYO manufacturers began to label loose tobacco as pipe tobacco, making these products available to RYO consumers at a lower price [10], [12]. As Morris showed, as soon as the tax rates changed, the amount of loose tobacco taxed as RYO declined dramatically, while the amount of loose tobacco taxed as pipe tobacco increased [10]. This practice was possible because, even though pipe tobacco and RYO tobacco traditionally have different physical characteristics (i.e. pipe tobacco is coarser and moister than RYO tobacco), for practical purposes the products are taxed and regulated according to the label on the packaging [12]–[14]. A lower price was realized because the Federal excise tax is paid by manufacturers who pass the cost to consumers through the final retail price. Additionally, because most states levy ad valorem taxes on pipe and RYO tobacco (i.e. taxes as a percentage of the product's overall price) [15], a lower Federal tax ultimately reduces states' excise and sales tax collections for tobacco products as well.

Loose tobacco labeled as pipe tobacco is being offered to consumers for making cigarettes. For example, starter kits are being sold that include a table-top injector machine, a box of cigarette tubes, and a bag of loose tobacco labeled “pipe tobacco" [16]. In addition, tobacco retailers in some states are offering customers the use of commercial cigarette rolling machines that can produce the equivalent of one carton of traditional cigarettes (i.e. 200 cigarettes) in approximately 8 minutes [17]. By using loose tobacco labeled as pipe tobacco, cigarettes produced by these machines are less expensive than factory-made cigarettes or cigarettes made from tobacco labeled as RYO [18]–[21].

Sellers of make-your-own cigarettes supplies use a range of terms to describe their products, including “dual purpose tobacco", “dual use tobacco" or “multi-use tobacco." This terminology helps prevent taxation of loose tobacco at the RYO rates. One online retailer posted “*This dual purpose tobacco is a highly recommended low-cost alternative to the standard cigarette tobacco. ‘Dual Purpose Tobacco’ is also called ‘Alternative Tobacco’ and ‘Pipe Cut Tobacco.’ ‘Pipe-cut’ pipe tobacco is the same as cigarette tobacco, with exception to the leaf being cut a little wider. Dual purpose pipe-cut tobacco is a dry tobacco works well with all of our cigarette machines and cigarette tubes*." [22]

This study quantifies the effect the Federal tax increase had on loose tobacco sales, and describes the policy and revenue implications of marketing pipe tobacco as “dual purpose" and selling it for RYO use, including estimating the total Federal and state revenue lost.

## Methods

Data on quantities of tobacco taxed in the United States between January 2007 and August 2011 come from monthly reports published by the Department of Treasury's Alcohol and Tobacco Tax and Trade Bureau (TTB) [23]. TTB collects Federal excise taxes on tobacco products that are intended for sale in the United States. State-specific pipe and RYO tobacco excise tax rates, sales tax rates on tobacco products, and cigarette sales volumes are from the Tax Burden on Tobacco [15].

Microsoft Excel 2010 and Adobe Illustrator CS3 were used to graph data. We used Joinpoint software to describe changes in loose tobacco sales trends (pipe tobacco plus RYO). The National Cancer Institute publishes Joinpoint software as a tool for assessing public health trends [24]. Joinpoint fits a segmented regression model to trend data, identifying the points where the segments meet and the trend changes (the “joinpoints") [25]. We specified a linear model assuming constant variance in the dependent variable.

To calculate revenue loses, TTB data were used to estimate the amount of loose tobacco marketed as pipe tobacco and sold for RYO use since the April 2009 federal tax change. In the 12 months prior to the tax increase, an average 432,000 pounds of pipe tobacco were taxed per month; this number is the baseline for comparison. For each month from April 2009 through August 2011, the difference between the amount of pipe tobacco taxed and the baseline amount was assumed to indicate the quantity of pipe tobacco sold for RYO use. The sum of the monthly differences is the cumulative amount (Equation 1).
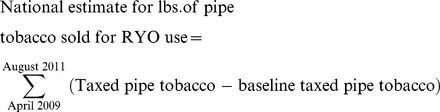
(1)


State-specific cigarette sales data are readily available, but few states report pipe tobacco sales data. To generate state-specific sales estimates for pipe tobacco sold for RYO use, we assumed that tobacco sales for RYO use were proportional to state cigarette sales [15]. We therefore used state cigarette sales data to establish the proportion of national cigarette sales that occurred in each state. These proportions were multiplied by the total estimated amount of pipe tobacco sold for RYO use nationally to get state-level estimates for each month. (Equation 2)
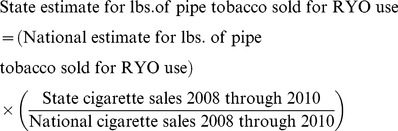
(2)


Most states levy the same excise tax rate on pipe and RYO tobacco, and base the tax on the wholesale or manufacturer's price for the product [15]. The manufacturer's price includes the federal tax, and after April 2009 the federal tax on pipe tobacco was $21.95/lb. lower than the tax on RYO tobacco [26]. Because loose tobacco sold for RYO use is less expensive at retail when it is taxed as pipe tobacco, it results in lower state excise and sales taxes being levied on the now less expensive product. Equation 3 shows the calculation for state excise tax losses used for most states. A similar calculation was used to estimate lost sales tax revenue.

Two states (ND and VT) tax RYO tobacco by the pound but tax pipe tobacco based on its price. For those states, we first calculated the amount of pipe tobacco sold for RYO use (Equation 2). We then calculated the total value of state excise tax for that amount of tobacco if it were taxed as RYO, then if it were taxed as pipe tobacco. The difference between the two totals represents the lost state excise tax revenue. Two states (AL and AZ) tax both pipe and RYO by the pound; for those states the difference in federal excise tax rates does not affect state excise tax collections, but does affect sales tax collections because sales taxes are based on price. 
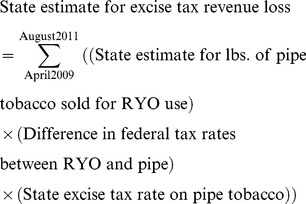
(3)


## Results

**Figure 1:**
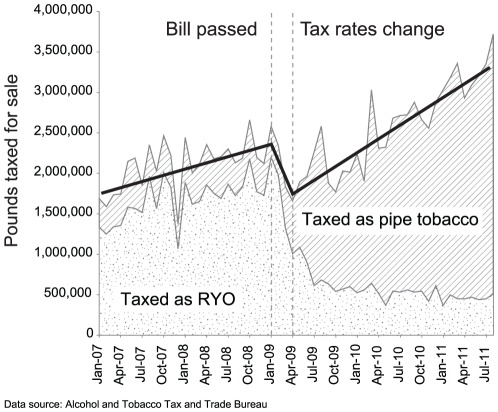
Roll-your-own (RYO) and pipe tobacco sales in the United States, January 2007–August 2011. This stacked area graph shows the total amount of loose tobacco (RYO and pipe tobacco) sales in the United States. The joinpoint fit line shows loose tobacco production was increasing by 15% annually prior to January 2009, mainly due to increases in RYO sales. Loose tobacco production dipped after the Federal tax increase was enacted, but only until the new tax rates went into effect in April 2009. Since April 2009, loose tobacco production has increased by 31% annually, twice as fast as before the tax was changed.

Joinpoint analysis identified two inflection points in the loose tobacco sales data: January 2009, when Congress passed the Federal tax increase (p<.001); and April 2009, when the tax changes took effect (p<.001) (Figure 1). The fit line on the figure shows loose tobacco production was increasing by 15% annually prior to January 2009, mainly due to increases in RYO sales. This is consistent with studies showing gradual increases in RYO use in the United States [9]. Loose tobacco production dipped after the Federal tax increase was enacted, but only until the new tax rates went into effect. Since April 2009, loose tobacco production has increased by 31% annually, twice as fast as before the tax was changed.

From April 2009 through August 2011, nearly 45 million pounds of pipe tobacco was sold for RYO use, lowering Federal excise tax collections by $985 million and lowering state sales and excise tax collections by more than $374 million (Table 2). When combined, over $1.36 billion has been lost in total state and Federal revenue as the result of this practice.

**Table 2:** $374 million in state revenue losses from RYO sold as pipe tobacco, April 2009–August 2011.

State	Revenue lost	State	Revenue lost
Florida	$63,090,040	Mississippi	$3,621,319
Texas	$31,230,931	Nevada	$3,593,092
California	$27,729,785	Connecticut	$3,155,720
New York	$16,949,733	Maryland	$2,948,031
Wisconsin	$16,648,628	Pennsylvania	$2,774,241
Oklahoma	$16,051,628	South Carolina	$2,738,305
Washington	$13,239,829	Rhode Island	$2,659,804
Minnesota	$13,049,342	Idaho	$2,453,330
Michigan	$12,718,593	Hawaii	$2,418,164
Ohio	$10,417,761	Utah	$2,029,489
Indiana	$10,083,434	Nebraska	$1,777,368
Arkansas	$9,679,677	West Virginia	$1,681,977
Illinois	$9,372,385	Montana	$1,524,148
Louisiana	$8,662,924	Alaska	$1,495,507
North Carolina	$7,737,982	Kansas	$1,353,789
Kentucky	$7,621,484	New Mexico	$1,267,201
Oregon	$7,505,630	Maine	$1,160,792
New Jersey	$6,937,258	Delaware	$1,005,273
Colorado	$6,044,448	South Dakota	$990,878
Iowa	$6,016,351	Alabama	$874,862
Massachusetts	$5,834,113	Wyoming	$632,761
Virginia	$5,430,809	Arizona	$627,551
Missouri	$5,257,990	North Dakota	$457,065
Georgia	$5,132,795	District of Columbia	$222,772
New Hampshire	$4,429,013	Vermont	$116,874
Tennessee	$3,954,684		

State revenue losses range from $63 million in Florida to $117,000 in Vermont. Eleven states have each lost over $10 million (CA, FL, IN, MI, MN, OH, NY, OK, TX, WA, WI), with lost revenue in those states accounting for 62 percent of all state revenue from RYO tobacco taxes lost.

## Discussion

The tax discrepancy between RYO and pipe tobacco offered an opportunity for tobacco manufacturers to lower the price consumers pay for loose tobacco used for making RYO cigarettes. Our analysis indicates that this approach led to a substantial increase in the sale of loose tobacco sold for RYO purposes, and in overall loose tobacco sales.

While rates of make-your-own cigarette use in the United States were increasing slowly before the tax change [9], the dramatic shift in sales after April 2009 can be partially explained by manufacturers labeling loose tobacco as pipe tobacco, allowing retailers to offer these products to RYO consumers at a lower price [12]. One factor that may have contributed to the sudden increase in RYO sales was the emergence of automated cigarette-rolling machines in retail stores.

Federal government and state government agencies have taken actions to attempt to curtail these tax revenue losses. For example, TTB, in its authority as the agency responsible for collecting Federal excise taxes, issued a ruling in September 2010 that found that retailers offering cigarette rolling machines are manufacturers of tobacco products, and are thus required to pay the Federal tax on all cigarettes that are produced [17]. Retailers sued TTB and a preliminary injunction was issued by the United States District Court for the Northern District of Ohio on December 14, 2010, preventing TTB from enforcing its ruling while the case remains pending [27]. As of March 2012 this court case was still pending. At the state level, New Hampshire's State Supreme Court ruled that by offering cigarette rolling machines, retailers would be classified as cigarette manufacturers and as a result would be subject to the Master Settlement Agreement, and be required to submit payments to the state for each cigarette that is produced [28]. Additionally, in March 2011, Arkansas enacted a law to prohibit licensed tobacco retailers from possessing or otherwise utilizing a cigarette rolling machine [29]. Also, the Wisconsin Department of Revenue issued a notice in September 2011 that ruled that retailers that offer cigarette rolling machines are classified as manufactures, and considers the final product to be a manufactured cigarette subject to cigarette excise taxes [30].

Selling pipe tobacco for RYO use avoids other laws and regulations as well. For example, the Prevent All Cigarette Trafficking (PACT) Act of 2009 prohibits the U.S. Postal Service from shipping cigarettes, RYO, and smokeless tobacco, but does not prohibit shipping pipe tobacco [31]. This allows internet sites to continue to sell and ship pipe tobacco marketed for RYO use. Further, the PACT Act requires sellers to report on quantities of cigarettes, RYO, and smokeless tobacco shipped to each state and tax administrators use this information to ensure all state taxes have been paid. There is no such reporting requirement on sales of pipe tobacco.

Additionally, the Family Smoking Prevention and Tobacco Control Act (Tobacco Control Act) prohibits candy-flavored cigarettes and RYO, but does not prohibit flavorings in pipe tobacco [32]. Brands of pipe tobacco sold for RYO use come in blackberry, black cherry, and vanilla flavors [22]. The Tobacco Control Act also prohibits the use of the descriptors “light," “mild," or “low," or similar descriptors in tobacco product labeling or advertising [32]. However, some pipe tobacco brands sold for RYO use still carry these descriptors [33].

This study has at least five limitations. First, we assumed that all pipe tobacco sales that exceeded the April 2009 baseline represented sales of pipe tobacco marketed for RYO use. This appears to be a reasonable assumption, given trends in pipe tobacco sales prior to the April 2009 tax increase. Second, for this study, the proportion of national cigarette sales that occur in each state is used as a proxy for the proportion of RYO tobacco sales in each state, causing actual RYO and pipe tobacco sales to vary from the estimates presented. This calculation also does not take into account different excise tax rates on non-cigarette tobacco products, which could further explain state-to-state variation in RYO tobacco use. Third, estimates do not factor in distributor or retailer markups. State excise and sales taxes are levied on products after these markups. Fourth, revenue lost estimates do not account for background trends in pipe tobacco sales prior to April 2009, although pipe tobacco sales were relatively flat during this period [23]. Finally, this study did not attempt to quantify changes in the number of taxed packs of cigarettes sold due to smokers switching from manufactured cigarettes to make-your-own cigarettes. Overall, these limitations mean our revenue loss estimates are likely conservative.

### Conclusion

Increasing excise taxes is one of the most effective evidence-based strategies for reducing tobacco use [1]–[4]. However, tax structures that provide tobacco users with an opportunity to switch to other low-cost tobacco products not only result in lower Federal and state revenue from these products, but also blunt the public health impact that excise tax increases would otherwise have on preventing youth initiation, reducing cigarette consumption and prompting quit attempts. In this instance, RYO and traditional cigarette smokers who may otherwise quit can instead maintain their addiction with lower priced products.
